# Unravelling the different causes of nitrate and ammonium effects on coral bleaching

**DOI:** 10.1038/s41598-020-68916-0

**Published:** 2020-07-20

**Authors:** Laura Fernandes de Barros Marangoni, Christine Ferrier-Pagès, Cécile Rottier, Adalto Bianchini, Renaud Grover

**Affiliations:** 1 Marine Department, Principality of Monaco, Centre Scientifique de Monaco, 8 Quai Antoine 1er, 98000 Monaco, Monaco; 2 Pós-Graduação Em Oceanografia Biológica, Oceanographic Institute, Federal University of Rio Grande, Av.Itália, Km 8, Rio Grande, RS 96203-900 Brazil; 3 Biological Science Institute, Federal University of Rio Grande, Av. Itália, Km 8, Rio Grande, RS 96203-900 Brazil

**Keywords:** Ecology, Physiology

## Abstract

Mass coral bleaching represents one of the greatest threats to coral reefs and has mainly been attributed to seawater warming. However, reduced water quality can also interact with warming to increase coral bleaching, but this interaction depends on nutrient ratios and forms. In particular, nitrate (NO_3_^−^) enrichment reduces thermal tolerance while ammonium (NH_4_^+^) enrichment tends to benefit coral health. The biochemical mechanisms underpinning the different bleaching responses of corals exposed to DIN enrichment still need to be investigated. Here, we demonstrated that the coral *Stylophora pistillata* underwent a severe oxidative stress condition and reduced aerobic scope when exposed to NO_3_^−^ enrichment combined with thermal stress. Such condition resulted in increased bleaching intensity compared to a low-nitrogen condition. On the contrary, NH_4_^+^ enrichment was able to amend the deleterious effects of thermal stress by favoring the oxidative status and energy metabolism of the coral holobiont. Overall, our results demonstrate that the opposite effects of nitrate and ammonium enrichment on coral bleaching are related to the effects on corals’ energy/redox status. As nitrate loading in coastal waters is predicted to significantly increase in the future due to agriculture and land-based pollution, there is the need for urgent management actions to prevent increases in nitrate levels in seawater. In addition, the maintenance of important fish stocks, which provide corals with recycled nitrogen such as ammonium, should be favoured.

## Introduction

Shallow-water coral reefs owe their ecological success in nutrient-poor tropical waters to the mutualistic symbiosis between corals and photosynthetic dinoflagellates of the family Symbiodiniaceae^1,2^. In addition to performing photosynthesis, these dinoflagellates take up dissolved inorganic nutrients such as nitrogen (nitrate, ammonium) and phosphorus from the surrounding water and recycle host metabolic wastes^3–6^. In shallow well-illuminated waters, 95% of the photosynthesis products can be translocated to the coral host^7,8^. These molecules support the holobiont (symbionts and host) metabolism, and contribute to coral growth, energy storage and reproduction^7,8^.

The coral-dinoflagellate association is particularly sensitive to thermal stress, with increasing temperatures resulting in the symbiosis breakdown, a phenomenon known as coral bleaching^9^. Many coral reefs are expected to be lost due to sea surface temperature rise as a result of global warming and increased frequency in pulse heat stress events, such as El Niño. Indeed, over the period 1871–2017, reefs have experienced unprecedented levels of thermal stress and mass bleaching^10–12^. In addition to this global stress, local stressors, such as eutrophication of coastal waters due to human activities, have also been linked to coral reef degradation^13^. In particular, elevated concentrations of dissolved inorganic nitrogen (DIN) in seawater have been associated to lower coral thermal tolerance, higher susceptibility to bleaching, prolonged bleaching and higher mortality^14,15^. However, the effects of increased DIN availability on coral bleaching are different depending on the nitrogen state (nitrate versus ammonium/urea), concentrations and phosphorus availability^14–16^. Nitrate enrichment has been shown to increase or prolong coral bleaching under thermal stress, especially with limited phosphorus availability^14,15,17^. More specifically, nitrate enrichment coupled with phosphorus starvation can result in changes in the algal lipid composition and destabilization of thylakoid membranes under increased light and temperature conditions^14^. On the other hand, moderate ammonium enrichment can be an important DIN source for maintaining photosynthesis and photosynthate translocation or calcification and delaying bleaching during thermal stress^18,19^. Yet, the complete physiological and biochemical mechanisms leading to the different thermal tolerance responses of corals when exposed to different DIN sources deserve further investigations.

Decades of research have suggested oxidants, including reactive oxygen and nitrogen species (ROS and RNS, respectively), as pivotal in the physiology of coral bleaching^20–25^. More specifically, an oxidative stress condition experienced by the coral holobiont under environmental stress, such as increasing temperature and high irradiance, is believed to be involved in the process of expulsion of symbionts by the coral host as a way to reduce the amount of ROS^22,23^. Oxidative stress takes place when prooxidants overwhelm the antioxidant mechanism, which depends on the level of ROS or/and RNS production, as well as strength of the defense and repair mechanism^22^. Oxidative stress can result in several cellular toxicity processes, such as damage to biomolecules, thus affecting cell functionality^26^. In addition, activation of cellular protection mechanisms involves a considerable energetic demand associated with reduced aerobic scope, which can result in deleterious effects on the whole-organism performance due to reduced energy flux^27,28^.

Nitrate and ammonium nutrition have been demonstrated to affect the oxidative metabolism of terrestrial plants in different ways. For instance, ammonium was shown to diminish oxidative stress in spinach plants, and to promote amino acid synthesis and protein turnover^29^. On the contrary, nitrate reductase (NR), which catalyzes the reduction of nitrate to nitrite, can lead to production of nitric oxide (NO) both in cultured *Symbiodiniaceae*^30^ and in plants submitted to stress^31^. Alternatively, plants and microalgae can produce NO through the mitochondrial enzyme activity or by the non-enzymatic reduction of apoplastic nitrite^32–34^. While NO is known to be a major mediator of physiological control, it can also be a critical amplifier of oxidative stress through its conversion to nitrating species, such as peroxynitrite^35^.

Considering the aforementioned effects on plants, it is possible to infer that nitrate and ammonium, once taken up by the coral holobiont, may influence its oxidative metabolism and its susceptibility to bleaching^22^, however, no study has been conducted so far in order to link DIN sources with coral physiology, oxidative status and bleaching. In fact, there is little evidence on the physiological and biochemical mechanisms involved in the contrasting effects of the different nitrogen states. Thus, in this work, we hypothesized that: (1) seawater enrichment with nitrate favors oxidative stress occurrence, contributing to a higher susceptibility of corals to bleaching under thermal stress conditions, (2) a more severe redox imbalance in corals facing nitrate enrichment and thermal stress poses extra-energy costs to the holobiont, and (3) ammonium enrichment has a protective effect on the oxidative response and energy metabolism by favoring amino acid synthesis and protein turnover.

In order to test these hypotheses, several parameters of oxidative and energy status (levels of ROS and RNS production, low molecular antioxidant defense system, oxidative damage occurrence and lactate concentration) were assessed in corals maintained under moderate ammonium and nitrate enrichment, then followed by thermal stress and recovery conditions. Physiological responses (symbiont density, chlorophyll content, protein biomass, maximal photosynthetic efficiency and calcification) were also quantified. The level of nitrate enrichment was 3 µM, which is a concentration that can be measured in eutrophicated reefs^36–39^. The level of ammonium enrichment was set at the same concentration, for comparison. Broadly, our results provide relevant and detailed insights into the different effects that these two DIN sources have on coral oxidative and energy metabolisms. More specifically, we were able to link oxidative stress, energy deficit and enhanced bleaching with nitrate enrichment only. On the contrary, ammonium enrichment, maintained the stability of the symbiosis under thermal stress by enhancing its energetic status.

## Material and methods

### Experimental set up

Five genetically different *Stylophora pistillata* colonies, originally sampled in the Gulf of Aqaba under the CITES number DCI/89/32 and then cultured at the Centre Scientifique de Monaco (CSM), were used to generate a total of 240 nubbins (48 per colony). Nubbins were attached to nylon threads, randomly distributed and kept into 12 independent 20-L experimental tanks supplied with natural seawater (flow rate of 10 L h^−1^). Metal halide lamps (Philips, HPIT 400 W, Distrilamp, Bossee, France) provided irradiance of 150 µmol photons m-^2^ s^−1^ (12:12 light:dark). Seawater temperature was kept at 25 ± 0.5 °C using submersible resistance heaters (Visi-ThermH Deluxe, Aquarium Systems, Sarrebourg, France) and salinity values were constant at 38 PSU. Submersible pumps ensured proper water mixing. Aquaria were cleaned weekly to avoid algal proliferation.

Coral nubbins were acclimated for 3 weeks in the experimental tanks and fed twice a week, at repletion, with *Artemia salina* nauplii. After acclimation, feeding was stopped and three nutritional treatments were implemented (Phase 1—Nitrogen enrichment, with four tanks per condition): (1) control condition (control), with natural seawater (ca. 0.5 µM DIN, 0.2 µM P); (2) NO_3_^−^ enriched condition (NO3), in which corals were exposed to a 3 µM NO_3_^−^ seawater enrichment; and (3) NH_4_—enriched condition (NH4), in which corals were exposed to a 3 µM NH_4_^+^ seawater enrichment. It’s important to note that the N:P ratio of the nitrogen enriched conditions (17:1) was closed to the Redfield ratio (16:1) and therefore, phosphorus was not a limiting nutrient compared to nitrogen. After 3 weeks under these conditions, thermal stress was implemented in half of the tanks of each nutritional condition, totalizing six treatments (Phase 2—Nitrogen enrichment + thermal stress, with two tanks per condition). Temperature was raised from 25 ± 0.5 °C to 30 ± 0.5 °C over a 7 day-period (0.7 °C per day) and maintained at 30 °C for another 7 days. After 2 weeks of thermal stress, all treatments were ceased and corals were kept in their respective aquaria for two more weeks under control conditions in order to evaluate their recovery (Phase 3—Recovery).

To perform nitrogen enrichment conditions in the tanks, stock solutions of nitrate (as NaNO_3_) and ammonium (as NH_4_Cl) were pumped at constant flow rate (0.3 L h^−1^) from a batch tank using peristaltic pumps (REGLO Digital, ISM 833, ISMATEC®). Nutrient concentrations (NO_3_^−^ and NH_4_^+^) in the experimental tanks were monitored weekly using an Autoanalyzer (Alliance Instrument, AMS, France) and remained constant in the tanks throughout the whole experiment. Coral samples for physiological and biochemical analysis were taken for each treatment at the end of each phase of the experiment as described below.

### Physiological measurements

#### Chlorophyll concentration, symbiont density, protein content and PAM fluorometry

Coral nubbins (n = 5 per treatment, Phase 1 = 15 nubbins, Phase 2 = 30 nubbins, Phase 3 = 30 nubbins) were frozen at − 80 °C for posterior determination of chlorophyll (*a* and *c*_*2*_), coral host protein content, and symbiont density, following Hoogenboom et al*.*^40^. Tissue was removed from the skeleton and collected in 10 mL of 0.45 µM FSW using an airbrush. The tissue slurry obtained was homogenized with a potter grinder. Symbiont density was quantified microscopically via replicate haemocytometer counts in 500 µL subsamples (Neubauer haemocytometer, Marienfeld, Germany). In turn, coral host protein content was assessed, based on Bradford^41^, using the Bradford Protein Assay Kit (23,200, Thermo Scientific, USA). Chlorophyll concentration was determined in accordance with Jeffrey and Humphrey^42^. For this purpose, 5 mL of of the homogenized tissue slurry was centrifuged (8,000 g, 10 min), the supernatant was removed, and the symbionts resuspended in 5 mL acetone for Chl *a* and *c*_*2*_ extractions. Data were normalized to surface area (cm^2^) using the wax-dipping method^43^. Maximum quantum yield of photosystem II (*F*_*v*_*/F*_*m*_,), which is correlated with photosynthetic efficiency of the symbionts, was assessed (n = 6 per treatment) using Pulse Amplitude Modulation (PAM) fluorometer (Walz, Germany), as a way to assess possible damages to photosystem II^44,45^. Minimal (F_0_) and maximal (F_m_) fluorescence yields were measured after dark acclimation (20 min) of coral nubbins, and measurements were performed at the same time of the day (11:00 am) during the whole experiment.

#### Calcification rates

Calcification rates were assessed on 5 coral nubbins per treatment (total of 30 nubbins) according to the buoyant weight technique^46^. Coral nubbins were hung on a nylon wire and weighed in seawater of known density using a Mettler XP205 electronic balance. Seawater temperature was controlled using a water bath (25 °C) and salinity continuously measured. Growth rates are presented as percent weight increase per week, using the following equation: [(*F* − *I*) / (*I* × number of weeks)] × 100, where *F* is the weight of each nubbin at the end of a given time step and *I* is the weight at the beginning of a given time step.

### Oxidative and energy metabolism analysis

#### Sample preparation

To perform the biochemical assays 5–4 nubbins were collected from each aquarium (Phase 1 = 27 nubbins, Phase 2 = 54 nubbins, Phase 3 = 54 nubbins). Subsequently, small coral pieces (N = 5–6 per treatment) were cut (0.5 cm^2^) and homogenized in ice by ultrasound (Frequency 70 kHz, Vibra-Cell™ 75,185, Bioblock Scientific, France) using 250–300 µl of the specific homogenization buffer for each analysis, as described below. After sonication, the remaining skeleton was discarded, the holobiont homogenized solution was centrifuged, and the intermediary phase was collected and immediately used for analysis. Total protein content of holobiont sample homogenates was determined according to Bradford^41^ using the “Comassie (Bradford) Protein Assay Kit” (23,200, Thermo Scientific, USA).

#### Reactive oxygen species (ROS) and nitric oxide (NO) detection

ROS quantification was performed using the fluorescent probe 5-(and-6)-carboxy-2′,7′-dichorodihydroflurescein (H_2_DCFDA, Molecular Probes) in line with Aguiar et al.^47^, with some modifications. Briefly, samples were homogenized in a buffer containing Tris–HCl 100 mM (pH 7.75), EDTA 2 mM, and MgCl_2_ 5 mM, and centrifuged for 20 min (20,000* g*, 4 °C). Samples protein content was adjusted to a final concentration of 0.5 mg/mL and 20 µL was added in a flat-bottom black microplate containing the following medium: HEPES 30 mM, KCl 200 mM and MgCl_2_ 1 mM (pH 7.2). Finally, 10 µL of H_2_DCFDA 16 µM was added and the fluorescence (excitation: 488 nm; emission: 525 nm) was measured every 5 min up to 50 min using a spectrofluorometer (Xenius®, SAFAS, Monaco). NO was quantified in the same way, however, the probe 4-Amino-5-Methylamino-2`,7`-Difluoroflurescein Diacetate (DAF-FM Diacetate, Invitrogen) was used instead. The results were expressed as fluorescence units per min (F.U. x min).

#### Lipid peroxidation (LPO)

LPO is one of the most prevalent mechanisms of cellular injury and has been extensively reported for marine organisms under oxidative stress condition^22^. LPO quantification was performed according to Oakes & van der Kraak^48^. Briefly, samples were homogenized in KCl (1,15%) solution containing 35 µM butylatedhydroxytoluene (BHT) and centrifuged for 10 min (10,000* g*, 4 °C). This method is based on the 2-thiobarbituric acid reactive substances (TBARS), and quantifies the peroxidative damage to lipids through the reaction between malondialdehyde (MDA), a byproduct of lipid peroxidation, and thiobarbituric acid (TBA). The reaction, at high temperature and acidity, generates a chromogen that is measured by spectrofluorometry (excitation: 515 nm; emission: 553 nm). Measurements were performed in a 96-well flat-bottom black microplate using a spectrofluorometer (Xenius®, SAFAS, Monaco). Data was normalized considering the total protein content in the samples homogenates in each well and expressed as nmol MDA mg protein^−1^.

#### Protein tyrosine nitration (PTN)

3-nitrotyrosine modification, a well-established marker of protein damage, is a product of PTN resulting from oxidative damage to proteins by peroxynitrite which is formed in vivo by the reaction of nitric oxide and superoxide anion^35^. The level of nitrotyrosine-modified proteins in corals was determined using the commercial kit “Nitrotyrosine ELISA Kit” (ab113848 Abcam) following manufacturer’s instructions. Briefly, the provided microplates are evenly coated with a nitrotyrosine containing antigen, and competitive ELISA is performed by adding the test sample mixed with the provided HRP conjugated anti-3NT antibody. A standard curve is generated from provided standard (3NT BSA standard) for accurate quantification of the 3NT content in the test samples. The assay was followed by monitoring the HRP-dependent color change in each well at 600 nm using a spectrofluorometer (Xenius®, SAFAS, Monaco). Data were normalized considering the total protein content in the sample homogenates in each well and expressed as ng NT-BSA mg protein^−1^.

#### Total antioxidant capacity (TAC)

Measuring organisms overall oxyradical scavenging capacity (hereafter referred as total antioxidant capacity—TAC) provides a more integrated assessment of susceptibility of a tissue/organism to oxidative stress^49^. Corals TAC was determined using the “OxiSelectTM Total Antioxidant Capacity (TAC) Assay Kit” (Cell Biolabs Inc., San Diego, CA, USA) according to manufacturer’s instructions. This assay measures the total antioxidant capacity of biomolecules via single electron transfer (SET) mechanism^49^, and it is based on the reduction of copper (II) to copper (I) by antioxidants, with marginal radical interference. Upon reduction, the copper (I) ion further reacts with a coupling chromogenic reagent with a maximum absorbance at 490 nm. The net absorbance values of antioxidants of coral holobiont samples were compared with a known uric acid standard curve, with absorbance values being proportional to the sample´s total reductive capacity. Absorbance readings were performed in a 96-well flat-bottom transparent microplate using spectrofluorometer (Xenius®, SAFAS, Monaco). Data were normalized considering the total protein content in the sample homogenates in each well and expressed as µM Copper Reducing Equivalents (CRE) mg protein^−1^.

#### Lactate concentration

Aquatic invertebrates under stressful conditions can undergo reduced aerobic scope and consequential increase in production of lactate, which can be used as a bioenergetic marker^50^. L( +)-Lactate was quantified in coral samples using the enzymatic assay “Lactate Assay Kit” (MAK064, Sigma-Aldrich, USA), which results in a colorimetric (570 nm) product proportional to the lactate present in the sample homogenates. Samples were deproteinized with a 10 kDa Molecular Weight Cut-off (MWCO) Spin Filter prior analysis to avoid LDH activity to degrade lactate. Data is presented as Lactate concentration (ng/µL).

### Data presentation and analysis

All data are expressed as mean ± standard error. We first tested the tank effect according to the procedure described in Underwood^51^, to check that variation among experimental units was zero, and that pooling was appropriate and did not change the conclusion of the analysis. As there was no tank effect, the tanks were pooled and the effects of nitrogen enrichment (as NH_4_^−^ and NO_3_^+^ addition) and increasing temperature on the physiological and biochemical parameters were evaluated using one-way analysis of variance (ANOVA) for data obtained at *Phase 1* (nitrogen enrichment) and *Phase 3* (recovery) of the experiment. In turn, data from *Phase 2* (nitrogen enrichment + thermal stress) were evaluated using two-way ANOVA. Data were checked for normality using the Shapiro–Wilk test and Homoscedasticity using Levene´s test. Data were log-transformed to meet ANOVA assumptions when necessary. If indicated, ANOVA were followed by the Student–Newman–Keuls (SNK) test. Differences were considered significant when *p* ≤ 0.05.

## Results

Significant differences in parameters were observed between treatments and experimental phases, except for total protein content that did not significantly vary throughout the entire experiment (data not shown). Details on the statistical results and the effect size in relation to the control in each of the experimental phases are presented in Tables 1, 2 and 3.

**Table 1:** Summary of one-way ANOVAs for the physiological [chlorophyll content, symbiont density, protein content, photosynthetic efficiency (*F*_*v*_*/F*_*m*_), calcification rates] and biochemical [reactive oxygen species (ROS) and nitric oxide (NO) production, total antioxidant capacity (TAC), lipid peroxidation (LPO), protein tyrosine nitration (PTN), lactate concentration] parameters after corals exposure to nitrogen enrichment (*Phase 1* of the experiment) and recovery (Phase 3). Significant *p* values (*p* ≤ 0.05) are in bold.

Parameters	Phase 1			Phase 3		
	*df*	*F*	*p*	*df*	*F*	*p*
Chlorophyll	2	5.56	**0.02**	5	3.38	**0.02**
Symbiont density	2	2.94	0.08	5	3.97	**0.01**
Protein content	2	0.89	0.43	5	2.43	0.07
Fv/Fm	2	11.80	**0.001**	5	1.82	0.15
Calcification	2	5.65	**0.02**	5	6.04	** < 0.001**
ROS	2	17.61	** < 0.001**	5	4.45	**0.002**
NO	2	12.52	** < 0.001**	5	5.21	**0.001**
TAC	2	4.51	**0.02**	5	5.93	** < 0.001**
LPO	2	8.92	**0.01**	5	0.80	0.56
PTN	2	3.95	**0.05**	5	10.72	** < 0.001**
Lactate	2	0.25	0.78	5	4.15	**0.02**

**Table 2:** Summary of two-way ANOVA for the physiological and biochemical parameters after coral exposure to nitrogen enrichment and thermal stress (Phase 2 of the experiment). Significant *p* values (*p* ≤ 0.05) are in bold.

Parameters	Effect	Phase 2		
		*df*	*F*	*p*
Chlorophyll	DIN	2	10.88	** < 0**.**001**
	Temp	1	43.96	** < 0**.**001**
	DIN*Temp	2	0.005	0.994
Symbiont density	DIN	2	16.27	** < 0**.**001**
	Temp	1	73.09	** < 0**.**001**
	DIN*Temp	2	0.95	0.398
Protein conten	DIN	2	3.19	0.071
	Temp	1	0.35	0.559
	DIN*Temp	2	0.64	0.533
Fv/Fm	DIN	2	3.92	**0**.**035**
	Temp	1	57.2	** < 0**.**001**
	DIN*Temp	2	5.1	**0**.**015**
Calcification	DIN	2	7.467	**0**.**004**
	Temp	2	3.77	**0**.**041**
	DIN*Temp	4	0.88	0.48
ROS	DIN	1	21.11	** < 0**.**001**
	Temp	2	2.41	0.099
	DIN*Temp	2	0.32	0.726
NO	DIN	1	3.96	**0**.**05**
	Temp	2	13.05	** < 0**.**001**
	DIN*Temp	2	1.28	0.296
TAC	DIN	1	2.6	0.115
	Temp	2	10.77	** < 0**.**001**
	DIN*Temp	2	0.87	0.425
LPO	DIN	1	26.837	** < 0**.**001**
	Temp	2	6.72	**0**.**005**
	DIN*Temp	2	6.86	**0**.**004**
PTN	DIN	1	59.71	** < 0**.**001**
	Temp	2	12.31	** < 0**.**001**
	DIN*Temp	2	4.69	**0**.**021**
Lactate	DIN	2	19.35	** < 0**.**001**
	Temp	1	17.64	** < 0**.**001**
	DIN*Temp	2	10.79	** < 0**.**001**

**Table 3:** Effect size (%) on the physiological and biochemical parameters relative to the control condition in each of the experimental phases (Phase1: nitrogen enrichment; Phase 2: nitrogen enrichment + thermal stress; Phase 3: recovery). (↑) indicates increased levels, (↓) indicates decreased levels, and (–) indicates non-significant changes.

Order of exposure	Treatments	Effect size (%) on parameters relative to control									
		Symbionts	Chl	Fv/Fv	Calcification	ROS	NO	TAC	LPO	PN	Lactate
Phase 1	NO_3_	–	–	↓21.4	↓34.8	↑30.0	↑55.9	↑28.1	↑49.9	↑43.6	–
	NH_4_	–	↑57.5	–	–	↓26.6	–	–	–	–	–
Phase 2	NO_3_	–	–	–	–	–	↑51.5	↑21.6	–	–	–
	NH_4_	↑50.4	↑51.2	–	↑51.3	–	–	–	↓78.6	↓61.4	↓80.5
	Temp	↓45.8	↓36.3	↓30.8	↓65.7	↑54.3	–	↑19.1	–	↑41.8	–
	Temp + NO_3_	↓33.4	↓28.4	↓42.3	–	↑58.3	↑47.8	↑26.8	↑150.0	↑78.0	↑119.2
	Temp + NH_4_	–	–	↓23.1	–	–	–	–	–	–	–
Phase 3	NO_3_	–	↓57.6	–	–	–	–	↑70.5	–	–	–
	NH_4_	–	–	–	–	–	–	–	–	–	↓65.9
	Temp	↓53.0	↓61.0	–	↓59.5	–	–	↑74.5	–	–	–
	Temp + NO_3_	↓50.2	↓65.1	–	↓98.5	↑90.3	↑96.3	↑68.5	–	↑82.6	–
	Temp + NH_4_	–	–	–	↓79.4	–	–	–	–	–	–

### Physiological parameters

**Figure 1:**
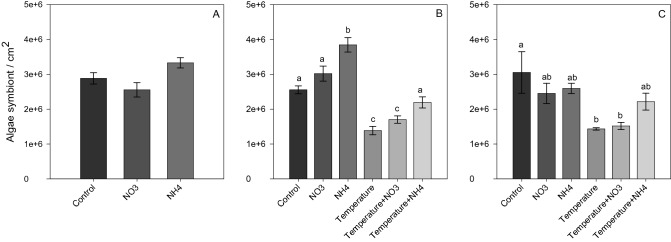
Symbiont density (algae symbiont/cm^2^) of *Stylophora pistillata* at (**A**) *Phase 1*—nitrogen enrichment, (**B**) *Phase 2*—nitrogen enrichment and thermal stress (isolated and combined treatments) and (C) *Phase 3*—recovery. Data are expressed as mean ± standard error. Different lowercase letters indicate significant differences among treatments (*p* < 0.05).

**Figure 2:**
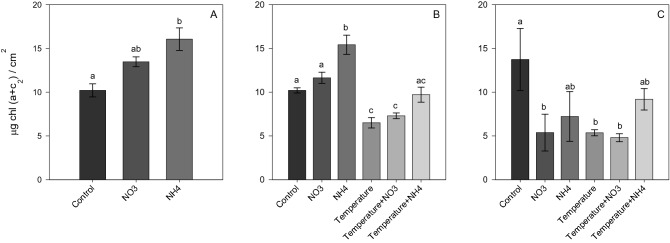
Total chlorophyll content (µg Chl (a + c_2_) / cm^2^) of *Stylophora pistillata* at (**A**) *Phase 1*—nitrogen enrichment, (**B**) *Phase 2*—nitrogen enrichment and thermal stress (isolated and combined treatments) and (**C**) *Phase 3*—recovery. Data are expressed as mean ± standard error. Different lowercase letters indicate significant differences among treatments (*p* < 0.05).

**Figure 3:**
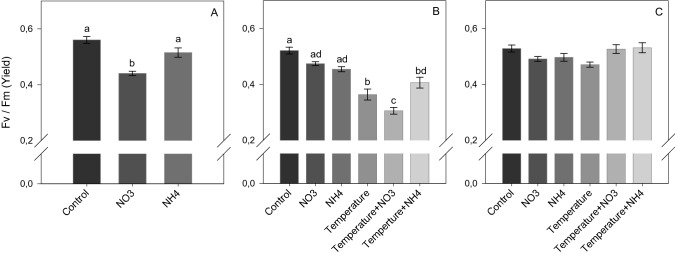
Maximal photosynthetic efficiency of photosystem II (*F*_*v*_*/F*_*m*_) in *Stylophora pistillata* at (**A**) Phase 1—nitrogen enrichment, (**B**) *Phase 2*—nitrogen enrichment and thermal stress (isolated and combined treatments) and (**C**) Phase 3—recovery. Data are expressed as mean ± standard error. Different lowercase letters indicate significant differences among treatments(*p* < 0.05).

**Figure 4:**
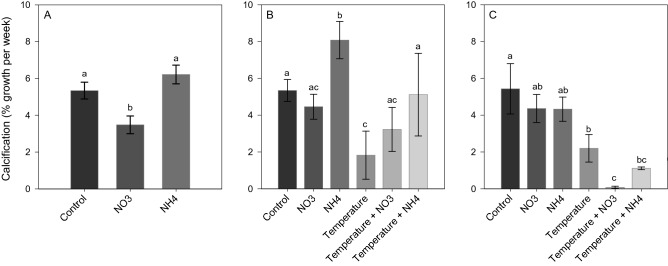
Calcification rates (% growth per week) in *Stylophora pistillata* at (**A**) *Phase 1*—nitrogen enrichment, (**B**) *Phase 2*—nitrogen enrichment and thermal stress (isolated and combined treatments) and (**C**) *Phase 3*—recovery. Data are expressed as mean ± standard error. Different lowercase letters indicate significant differences among treatments (*p* < 0.05).

Nitrate enrichment effect: At *Phase 1*, NO_3_ enrichment did not lead to significant changes in the symbiont density and chlorophyll content compared to other treatments (Figs. 1A and 2A), but induced a significant decrease in the maximal photosynthetic efficiency of PSII (*F*_*v*_*/F*_*m*_) (SNK, *p* < 0.001; Fig. 3A) and in calcification rates (SNK, *p* < 0.04; Fig. 4A). At *Phase 2*, corals exposed to increased temperature alone, or in combination with NO_3_ enrichment, experienced a significant decrease in symbiont density (SNK, *p* < 0.02; Fig. 1B) and chlorophyll content (SNK, *p* < 0.04; 2B) compared to other treatments. The combined treatment of thermal stress and NO_3_ enrichment also caused a significant decrease in photosynthetic efficiency with respect to all other conditions tested (SNK, *p* < 0.03; Fig. 3B). In addition, increased temperature alone showed lower photosynthetic efficiency compared to all conditions maintained at 25 °C (SNK, *p* < 0.004; Fig. 3B), as well as lower calcification rates with respect to the control and both NH_4_ enriched conditions (SNK, p*p*< 0.05; Fig. 4B). After recovery (*Phase 3*), the photosynthetic efficiency of corals returned to control levels in all treatments (Fig. 3C), however, corals exposed to increasing temperature alone or combined to NO_3_ enrichment still kept lower symbiont density and chlorophyll content (SNK, *p* < 0.05; Fig. 2C) as well as a lower calcification rates (SNK, *p* ≤ 0.05; Fig. 4C) compared to those maintained under control condition. Corals exposed to the combined treatment of increasing temperature and NO_3_ enrichment were no longer able to calcify, with mean growth rates close to zero (Fig. 4C).

Ammonium enrichment effect: during *Phase 1*, chlorophyll content significantly increased in corals maintained under NH_4_ enrichment as regards to those under control condition (SNK, *p* = 0.01; Fig. 2A). In contrast to NO_3_ enrichment, there was no significant change in the *F*_*v*_*/F*_*m*_ and calcification rates compared to control corals (Figs. 3A and 4A). At *Phase 2*, corals exposed to NH_4_ enrichment and maintained at 25 °C still showed increased chlorophyll content (SNK, *p* < 0.04; Fig. 2B), as well as a significant increase in symbiont density (SNK, *p* < 0.02; Fig. 1B) (not observed in *Phase 1*) compared to all other treatments. The latter significant increase in symbionts density observed highlights that longer exposure to NH_4_ can have relevant effects. Also, and in contrast to nitrate, the combined treatment of elevated temperature and ammonium enrichment maintained similar symbiont density and chlorophyll content as the control condition (SNK, *p* > 0.05; Figs. 1C and 2C), and a higher photosynthetic efficiency compared to the combined temperature and nitrate condition (SNK, *p* < 0.004; Fig. 3B). After recovery (*Phase 3*), symbiont density and chlorophyll content in the combined treatment of temperature and ammonium were still similar to the control condition (SNK, *p* > 0.05; Figs. 1C and 2C). However, lower growth rates were observed in corals maintained in the combined treatment of increasing temperature and NH_4_ enrichment with respect to corals maintained under control condition (SNK, *p* = 0.02; Fig. 4C).

### Oxidative stress and energy metabolism parameters

The biochemical response of corals (oxidative stress and energy metabolism) was different between NO_3_ and NH_4_ enrichment treatments (Table 1 and 2).

**Figure 5:**
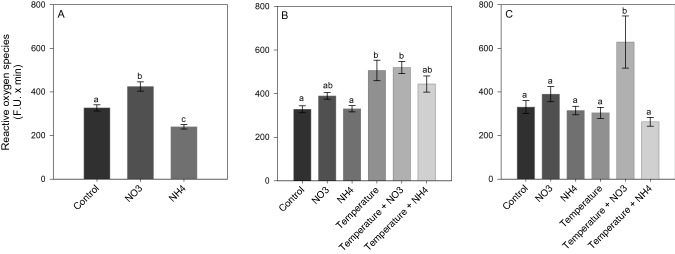
Reactive oxygen species production (F.U. × min) in *Stylophora pistillata* at (**A**) *Phase 1*—nitrogen enrichment, (**B**) *Phase 2*—nitrogen enrichment and thermal stress (isolated and combined treatments) and (**C**) *Phase 3*—recovery. Data are expressed as mean ± standard error. Different lowercase letters indicate significant differences among treatments (*p* < 0.05).

**Figure 6:**
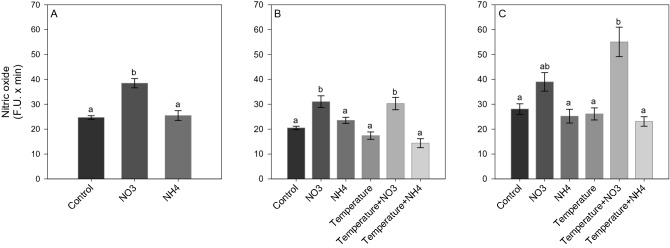
Nitric oxide production (F.U. × min) in *Stylophora pistillata* at (**A**) *Phase 1*—nitrogen enrichment, (**B**) *Phase 2*—nitrogen enrichment and thermal stress (isolated and combined treatments) and (**C**) *Phase 3*—recovery. Data are expressed as mean ± standard error. Different lowercase letters indicate significant differences among treatments (*p* < 0.05).

**Figure 7:**
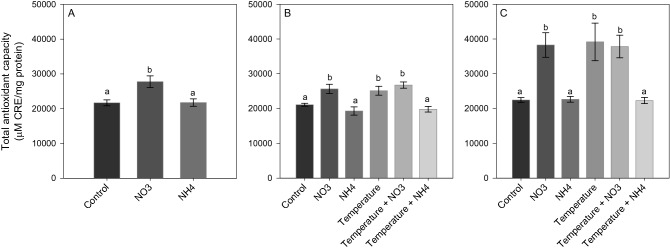
Total antioxidant capacity (TAC) (µM CRE/mg protein) of *Stylophora pistillata* at (**A**) *Phase 1*—nitrogen enrichment, (**B**) *Phase 2*—nitrogen enrichment and thermal stress (isolated and combined treatments) and (**C**) *Phase 3*—recovery. Data are expressed as mean ± standard error. Different lowercase letters indicate significant differences among treatments (*p* < 0.05).

**Figure 8:**
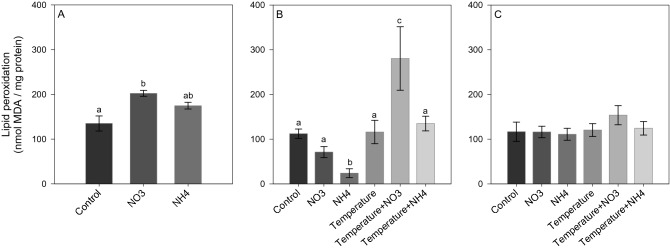
Lipid peroxidation (LPO) (nmol MDA/mg protein) in *Stylophora pistillata* at (**A**) *Phase 1*—nitrogen enrichment, (**B**) *Phase 2*—nitrogen enrichment and thermal stress (isolated and combined treatments) and (**C**) *Phase 3*—recovery. Data are expressed as mean ± standard error. Different lowercase letters indicate significant differences among treatments (*p* < 0.05).

**Figure 9:**
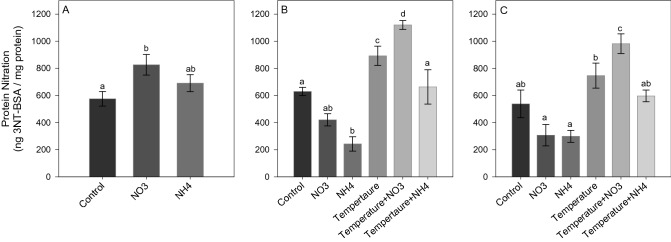
Protein tyrosine nitration (PTN) (ng 3NT-BSA/mg protein) in *Stylophora pistillata* at (**A**) *Phase 1*—nitrogen enrichment, (**B**) *Phase 2*—nitrogen enrichment and thermal stress (isolated and combined treatments) and (**C**) *Phase 3*—recovery. Data are expressed as mean ± standard error. Different lowercase letters indicate significant differences among treatments (*p* < 0.05).

**Figure 10:**
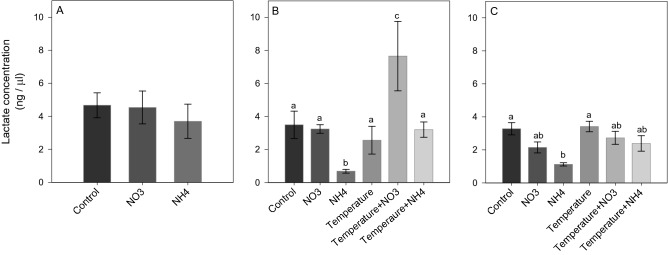
Lactate concentration (ng/µl) in *Stylophora pistillata* at (**A**) *Phase 1*—nitrogen enrichment, (**B**) *Phase 2*—nitrogen enrichment and thermal stress (isolated and combined treatments) and (**C**) *Phase 3*—recovery. Data are expressed as mean ± standard error. Different lowercase letters indicate significant differences among treatments (*p* < 0.05).

After *Phase 1* of the experiment, NO_3_ enrichment led to a significant increase in ROS and NO production (SNK, *p* < 0.02; Figs. 5A and 6A), as well as in TAC level (SNK, *p* < 0.04; Fig. 7A) compared to control and NH_4_ enriched corals. Also, an increase in LPO and PN was detected in corals under NO_3_ enrichment compared to those maintained in control condition (SNK, *p* < 0.04; Figs. 8A and 9A). Lactate concentrations did not show any differences among treatments (Fig. 10A). At *Phase 2*, corals exposed to NO_3_ enrichment alone, or combined with thermal stress, showed higher levels of NO with respect to all other treatments (SNK, *p* < 0.04; Fig. 6B). In turn, corals exposed to increased temperature alone, or combined with NO_3_ enrichment, presented increased ROS production compared to the control and NH_4_ enriched conditions (SNK, *p* < 0.02; Fig. 5B), and increased TAC values compared to all other conditions tested (*p* < 0.05; Fig. 7B), except for the NO_3_ enrichment condition alone. Finally, corals exposed to the combined treatment of NO_3_ enrichment and thermal stress showed higher LPO, PTN and lactate levels, compared to all other treatments (SNK, *p* < 0.04: Figs. 8B, 9B and 10B). After recovery (*Phase 3*), corals exposed to NO_3_ enrichment still presented higher levels of ROS and NO production (SNK, *p* < 0.04; Figs. 5C and 6C), as well as higher PTN levels (SNK, *p* < 0.04; Fig. 9C). LPO levels returned to control levels in all treatments (Fig. 8C). TAC values remained higher in the NO_3_ enrichment and increasing temperature treatments applied alone or in combination, compared to all other conditions tested (SNK, *p* < 0.03. Figure 7C).

In contrast to NO_3_ enrichment, NH_4_ addition induced a significant decrease in ROS levels at *Phase 1* of the experiment (SNK, *p* < 0.02; Fig. 5A). At *Phase 2*, NH_4_ enriched corals presented lower levels of LPO (SNK, *p* < 0.04; Fig. 8B) and lactate concentration (SNK, *p* < 0.03, Fig. 10B) with respect to all other treatments. PTN was also lower compared to all treatments (SNK, *p* < 0.04; Fig. 9B), except for the NO_3_ enrichment condition. After recovery (*Phase 3*), ROS, NO, TAC and LPO levels in NH_4_ enriched corals under ambient and elevated temperature were the same observed for control corals. PTN levels in NH_4_ enriched corals also did not differ from control corals, however, those were significantly lower compared to corals exposed to elevated temperature alone, or combined to NO_3_ enrichment (SNK, *p* < 0.05; Fig. 9C). Corals exposed to NH_4_ enrichment and elevated temperature showed lower PTN levels compared to the ones exposed to NO_3_ enrichment and elevated temperature (SNK, *p* < 0.05; Fig. 9C). Also, NH_4_ enriched corals showed a significant lower lactate concentration compared to those under control and thermal stress conditions (SNK, *p* < 0.03; Fig. 10C).

## Discussion

Although nitrate and ammonium enrichment tend to have different effects on coral health^14,18,19^, there is little evidence on the physiological and biochemical mechanisms involved under their contrasting effects. Here, we demonstrate that corals underwent a severe oxidative stress condition and reduced aerobic scope when exposed to NO_3_^−^ enrichment combined with thermal stress. On the contrary, NH_4_^+^ enrichment was able to amend the deleterious effects of thermal stress by favoring the oxidative status and energy metabolism of the coral holobiont.

Under normal growth conditions (25 °C), a transient imbalance in coral’s redox status was observed under NO_3_^−^ enrichment, as was reflected in increased levels of reactive species (ROS and NO) and oxidative damage (LPO and PTN) measured after 3 weeks of nitrate enrichment. Concurrent with the increase of oxidative stress, calcification rates and photosynthetic efficiency of symbiont’s photosystem II decreased. Such effect on calcification and photosynthesis might partly be due to the increased oxidative stress, and partly because nitrate enrichment negatively impacts carbon assimilation and translocation^19^. After 5 weeks of enrichment however, most biochemical and physiological parameters returned to control levels, likely because increased TAC levels counteracted the oxidative damage resulting from ROS and NO overproduction. Regardless of TAC levels, NO production remained significantly higher under NO_3_^−^ enrichment compared to the other conditions. When NO_3_^−^ enrichment was stopped, increased TAC levels helped to neutralize NO overproduction that finally returned back to control levels. The maintenance of high levels of antioxidant capacity usually requires extra energy^52^, but in this study, no sign of reduced aerobic scope (as indicated by lactate concentration) was observed. Overall, our results show that NO_3_^−^ enrichment favored reactive species formation in coral tissues, especially NO. The exact mechanism explaining NO overproduction is still unclear, but may be related to the activity of the nitrate reductase, which might have generated NO under stressful conditions, as observed in plants^31^.

As opposed to NO_3_^−^, NH_4_^+^ enrichment did not cause an imbalance of coral’s redox status. After 5 weeks lower levels of oxidative damage compared to control conditions were observed, likely due to an enhancement of coral metabolism. Indeed, the particularly low concentrations of lactate, which is the main final product of the anaerobic metabolism pathway^28^, observed after 5 weeks of NH_4_^+^enrichment and after recovery, suggest that the aerobic pathway was favored. Since the latter is known to be the most efficient energetically^28^, corals may have had greater energy resources to invest in antioxidant defenses (known to be energetically costly), and to fight against oxidative damage. Ammonium enrichment was shown to have several other benefits for corals such as an enhancement (1) in the rate of molecule repair through increased nucleic acid synthesis and protein turnover^18,29^; and (2) in photoprotective pigments^18^ that can contribute to membrane protection and consequently lower lipid peroxidation^53,54^.

As shown in previous work^55–57^, thermal stress alone induced oxidative stress in corals, which was evidenced by increasing ROS, TAC and PTN. In accordance, physiological measurements in our study indicated the onset of coral bleaching (decreased symbiont density, chlorophyll *a* content and photosynthetic efficiency) followed by decreased calcification rates. After recovery, ROS production returned to control levels, indicating that the increased TAC was able to neutralize ROS overproduction resulting from thermal stress. However, high levels of oxidative damage, together with decreased symbiont density, chlorophyll content and calcification rates were still observed. Considering that no major alterations in the energy metabolism were detected, as indicated by stable lactate concentrations throughout the whole experiment, energy resources may have been employed to neutralize the ROS overproduction rather than investment into physiological processes.

**Figure 11:**
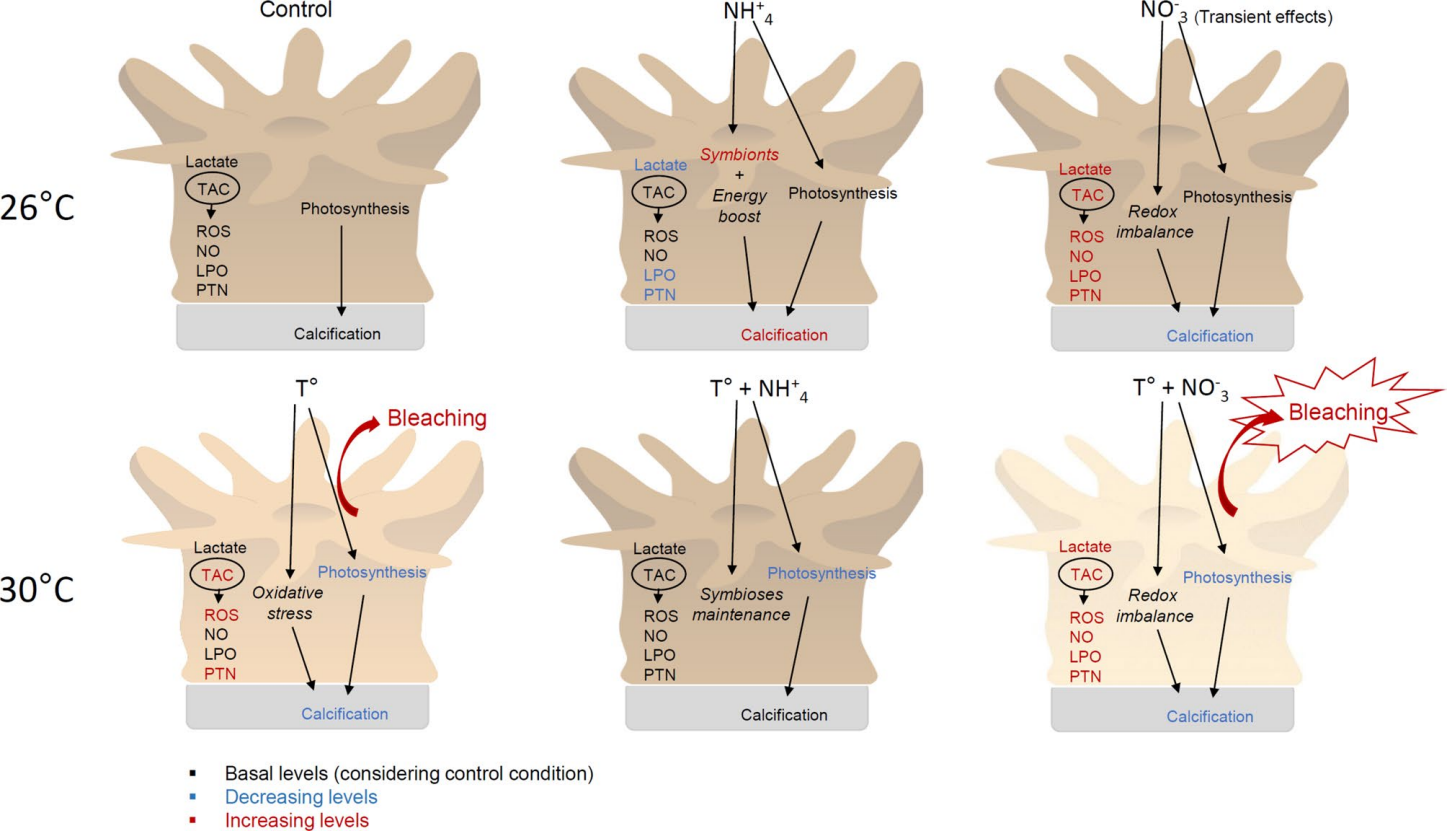
Conceptual model on the biochemical mechanisms involved in the contrasting coral bleaching responses to different sources of dissolved inorganic nitrogen [nitrate (NO_3_^−^) and ammonium (NH_4_^+^)] under thermal stress. Moderate seawater enrichment with nitrate leads to a transient imbalance in coral’s redox status, while ammonium benefits their energy metabolism. Nitrate enrichment under thermal stress significantly decreases coral’s aerobic scope and increases oxidative stress condition and bleaching response compared to a low-nitrogen condition. Ammonium enrichment under thermal stress maintains the stability of the symbiosis and does not lead corals to an oxidative stress condition.

The combination of NO_3_^−^ enrichment and thermal stress was highly detrimental to the health of corals, in accordance to former studies (for review see Morris et al*.*^58^). Our results however explained such effect by severe oxidative stress and reduced aerobic scope, as summarized in Fig. 11. This was highlighted by the overproduction of reactive species (ROS and NO) followed by significant increases of oxidative damage and TAC, as well as a two-fold increase in lactate concentration compared to control conditions. Information on how the energetic pathway of corals responds to stressors is lacking, however, it is worth noting that switching from aerobic to less efficient anaerobic metabolism has been linked to increased lipid peroxidation in other invertebrates, such as gastropods^59^. Increased lactate concentration can indeed be an indirect evidence of destructive processes in mitochondrial membranes, since damaged membranes are not able to promote a H^+^-gradient necessary for ATP synthesis. In this case, pyruvate converts into lactate to release the hydrogen carrier (such as FAD^+^ and NAD^+^) necessary for glycolysis^59–61^. It is worth noting, that NO, through peroxinitrite formation, can also inhibit mitochondrial NADH-ubiquinone reductase, which indirectly disturbs ATP generation^62^. Also, NO can decrease fatty-acid biosynthesis and interfere in amino-acid metabolism in coral tissues^63^.

After NO_3_^−^ enrichment and thermal stress were stopped, the continuous maintenance of high levels of TAC (compared to control corals) during recovery was not sufficient to reestablish ROS, NO and PTN back to control levels, and symbiont density and chlorophyll content also remained low compared to the other conditions. Further, the fact that corals were not able to calcify anymore is strong evidence that they were facing an energy deficit. Environmental stress can strongly affect energetic balance of organisms due to additional energy requirements and the allocation to different functions being fundamental to maintain homeostasis. Protein synthesis, for example, can be energetically demanding^28^. In this context, it is possible to infer that a substantial amount of energy was preferentially allocated to the activation of enzymatic and non-enzymatic antioxidant defenses to counteract oxidative stress. In this context, additional evidence has been provided by Higuchi et al*.*^64^ who demonstrated that superoxide dismutase and catalase activities can increase due to high temperature and nitrate enrichment in the coral *Montipora digitata*. Also, it is important to consider that corals acquire most of their energy through photosynthates translocated by the algal symbionts to perform basic physiological processes such as calcification^65^. Thus, reduced symbiont density may have led to a lower amount of energy available for the coral host, contributing to effects of metabolic energy deficit. Additionally, phosphate limitation (relative to nitrate) can lead to phosphorus competition within the holobiont with algal symbionts retaining nutrients and potentially sequestering ATP from their hosts^58^.

In contrast to NO_3_^−^, NH_4_^+^ enrichment mitigated the negative effects of thermal stress on coral physiology (Fig. 11), likely due to increased carbon acquisition and translocation to the host compared to control conditions^66^. In spinach plants, ammonium enrichment also stimulates amino acid content and production of phenolic compounds known as important antioxidants^29^. It’s also important to note that, after 5 weeks of NH_4_^+^ enrichment a significant increase in symbiont density was observed compared to control, however, when thermal stress was applied, symbiont density returned back to control levels. Such loss of symbionts can be related to the fact that excess algal symbionts under stressful conditions may generate more ROS on a per-cell basis^67^. Thus, maintaining normal algae density (same as control condition) may favor homeostasis in such conditions. In turn, the lower rates of calcification and photosynthetic efficiency observed after recovery can be explained by the fact that thermal stress impairs NH_4_^+^ assimilation in corals^19^. Thus, the equilibrium achieved during NH_4_^+^ enrichment followed by thermal stress and decreased concentrations of NH_4_^+^ during recovery may have caused a temporary imbalance in corals metabolism.

The present findings considerably advance our knowledge about the opposing effects of ammonium and nitrate on coral thermal tolerance. Our results are in agreement with previous evidence showing that NH_4_^+^ enrichment can enlarge symbiont populations whilst also increasing thermal tolerance in corals, and that NO_3_^−^ enrichment can increase bleaching susceptibility without prior symbiont density enlargement^16^. Our findings also agree with the assertion of Morris et al*.*^57^ who suggested that the different impacts of ammonium and nitrate on coral thermal tolerance can be explained by their relative effects on the carbon metabolism and oxidative stress of the coral holobiont. We indeed showed that while nitrate enrichment increases oxidative stress in corals, ammonium enrichment tends to decrease it. Taking into account the beneficial effects of ammonium on the oxidative and energy metabolisms observed here, future studies should determine the minimal amount of ammonium necessary to maintain the coral-Symbiodinaceae symbiosis during thermal stress. In addition, although corals were enriched with 3 µM nitrate or ammonium, the N:P ratio used in this study was still in the range of the Redfield ratio, meaning that the corals were not limited in phosphorus compared to the amount of nitrogen. Future studies should investigate whether the beneficial effects of ammonium observed here, are still conserved under an imbalance N:P ratio (sensu Wiedenmann et al. 2013, i.e. excess nitrogen compared to phosphorus). Finally, it would be worth investigating the extent by which NH_4_ enrichment can alleviate the negative effects of NO_3_ enrichment. Indeed, NO_3_ is the main form of nitrogen pollution released in coastal waters by anthropogenic activities such as agriculture, while ammonium is mainly recycled in water through fish excretion^68^. Therefore, urgent management actions should be taken to prevent increases in nitrate levels in seawater. Additionally, the maintenance of important fish stocks, which provide corals with recycled nitrogen such as ammonium, should be favoured.

## References

[1] Dubinsky Z, Jokiel PL (1994). Ratio of energy and nutrient fluxes regulates symbiosis between zooxanthellae and corals. Pac. Sci..

[2] LaJeunesse TC, Parkinson JE, Gabrielson PW, Jeong HJ, Reimer JD, Voolstra CR, Santos SR (2018). Systematic revision of Symbiodiniaceae highlights the antiquity and diversity of coral endosymbionts. Curr. Biol..

[3] Falkowski PG, Dubinsky Z, Muscatine L, Porter JW (1984). Light and the bioenergetics of a symbiotic coral. Bioscience.

[4] Grover R, Maguer J-F, Reynaud-Vaganay S, Ferrier-Pagès C (2002). Uptake of ammonium by the scleractinian coral *Stylophora pistillata*: effect of feeding, light, and ammonium concentrations. Limnol. Oceanogr..

[5] Grover R, Maguer J-F, Allemand D, Ferrier-Pagès C (2003). Nitrate uptake in the scleractinian coral *Stylophora pistillata*. Limnol. Oceanogr..

[6] Godinot C, Ferrier-Pagès C, Grover R (2009). Kinetics of phosphate uptake by the scleractinian coral *Stylophora pistillata*. Limnol. Oceanogr..

[7] Muscatine L, McCloskey LR, Marian RE (1981). Estimating the daily contribution of carbon from zooxanthellae to coral animal respiration. Limnol. Oceanogr..

[8] Trembley P, Grover R, Maguer J-F, Legendre L, Ferrier-Pagè C (2012). Autotrophic carbon budget in coral tissue: a new 13C-based model of photosynthate translocation. J. Exp. Biol..

[9] Hoegh-Guldberg O (2007). Coral reefs under rapid climate change and ocean acidification. Science.

[10] Claar DC, Szostek L, McDevitt-Irwin JM, Schanze JJ, Baum JK (2018). Global patterns and impacts of El Niño events on coral reefs: A meta-analysis. PLoS ONE.

[11] Lough JM, Anderson KD, Ughes TP (2018). Increasing thermal stress for tropical coral reefs: 1871–2017. Sci. Rep..

[12] Hughes TP (2018). Spatial and temporal patterns of mass bleaching of corals in the Anthropocene. Science.

[13] Lapointe BE, Brewton RA, Herren LW, Porter JW, Hu C (2019). Nitrogen enrichment, altered stoichiometry, and coral reef decline at Looe Key, Florida Keys, USA: a 3-decade study. Mar. Biol..

[14] Wiedenmann J, D’Angelo C, Smith EG, Hunt AN, Legiret F-E, Postle AD, Achterberg EP (2013). Nutrient enrichment can increase the susceptibility of reef corals to bleaching. Nat. Clim. Chang..

[15] Burkepile DE (2019). Nitrogen identity drives differential impacts of nutrients on coral bleaching and mortality. Ecosystems.

[16] Shantz AA, Burkepile DE (2014). Context-dependent effects of nutrient loading on the coral-algal mutualism. Ecology.

[17] Nordemar I, Nyströn M, Dizon R (2003). Effects of elevated seawater temperature and nitrate enrichment on the branching coral *Porites cylindrica* in the absence of particulate food. Mar. Biol..

[18] Béraud E, Gevaert F, Rottier C, Ferrier-Pagès C (2013). The response of the scleractinian coral Turbinaria reniformis to thermal stress depends on the nitrogen status of the coral holobiont. J. Exp. Biol..

[19] Ezzat L, Maguer J-F, Grover R, Ferrier-Pagès C (2015). Limited phosphorus availability is the Achilles heel of tropical reef corals in a warming ocean. Sci. Rep..

[20] Lesser MP (1996). Elevated temperatures and ultraviolet radiation cause oxidative stress and inhibit photosynthesis in symbiotic dinoflagellates. Limnol. Oceanogr..

[21] Lesser MP (1997). Oxidative stress causes coral bleaching during exposure to elevated temperatures. Coral Reefs.

[22] Lesser MP (2006). Oxidative stress in marine environments: biochemistry and physiological Ecology. Annu. Rev. Physiol..

[23] Downs CA, Fauth JE, Halas JC, Dustan P, Bemiss J, Woodley CM (2002). Oxidative stress and seasonal coral bleaching. Free Rad. Biol. Med..

[24] Perez S, Weis V (2006). Nitric oxide and cnidarians bleaching: an eviction notice mediates breakdown of a symbiosis. J. Exp. Biol..

[25] Weis VM (2008). Cellular mechanisms of Cnidarian bleaching: stress causes the collapse of symbiosis. J. Exp. Biol..

[26] Halliwell, B. & Gutteridge, J.M.C. (eds.) *Free Radicals in Biology and Medicine*. (Oxford, 2007). 10.1016/0748-5514(85)90028-5 3939136

[27] Pörtner HO, Farrell AP (2008). Physiology and climate change. Science.

[28] Sokolova IM (2013). Energy-Limited tolerance to stress as a conceptual framework to integrate the effects of multiple stressors. Integ. Comp. Biol..

[29] Dominguez-Valdivia MD, Aparacio-Tejo PM, Lamsfus C, Cruz C, Martins-Loução MA, Moran JF (2008). Nitrogen nutrtion and antioxidant metabolism in ammonium-tolerant and –sensitive plants. Phys. Plant..

[30] Bouchard JN, Yamasaki H (2008). Heat stress stimulates nitric oxide production in *Symbiodinium microadriaticum*: a possible linkage between nitric oxide and the coral bleaching phenomenon. Plant. Cell Physiol..

[31] Yamasaki H, Sakihama Y (2000). Simultaneous production of nitric oxide and peroxynitrite by plant nitrate reducatase: in vitro evidence for the NR*dependent formation of active nitrogen species. FEBS..

[32] Bethke PC, Badger MR, Jones RL (2004). Apoplastic synthesis of nitric oxide by plant tissues. Plant. Cell..

[33] Tischner R, Planchet E, Kaiser WM (2004). Mitochondrial electron transport as a source of nitric oxide in the unicellular green algae *Chlorella sorokiniana*. FEBS Lett..

[34] Planchet E, Gupta KJ, Sonoda M, Kaiser WM (2005). Nitric oxide emission from tabacco leaves and cell suspensions: rate limiting factors and evidence for the involvement of mitochondrial electron transport. Plant. J..

[35] Bartesaghi S, Radi R (2018). Fundamentals on the biochemistry of peroxynitrite and protein tyrosine nitration. Redox. Biol..

[36] Brodie J, Devlin M, Heynes D, Waterhouse J (2011). Assessment of the eutrophication status of the Great Barrier Reef lagoon (Australia). Biogeochemistry.

[37] Govers LL, Lamers LP, Bouma TJ, de Brouwer JH, van Katwijk MM (2014). Eutrophication threatens *Caribbean seagrass*: an example from Curaçao and Bonaire. Mar. Poll. Bull..

[38] Naumann MS, Bednarz VN, Ferse SC, Niggl W, Wild C (2015). Monitoring of coastal coral reefs near Dahab (Gulf of Aqaba, red sea) indicates local eutrophication as potential cause for change in benthic communities. Environ. Monit. Assess..

[39] Rouzé H, Lecellier G, Langlade M, Planes S, Berteaux-Lecellier V (2015). Fringing reefs exposed to different levels of eutrophication and sedimentation can support similar benthic communities. Mar. Pollut. Bull..

[40] Hoogenboom M, Beraud E, Ferrier-Pagè C (2010). Relationship between symbiont density and photosynthetic carbon acquisition in the temperate coral *Cladocora caespitosa*. Coral Reefs.

[41] Bradford MM (1976). A rapid and sensitive method for the quantitation of microgram quantities of protein utilizing the principle of protein-dye binding. Anal. Biochem..

[42] Jeffrey S, Humphrey G (1975). New spectrophotometric equations for determining chlorophylls a, b, c1 and c2 in higher plants, algae and natural phytoplankton. Biochem. Physiol. Pfl..

[43] Veal CJ, Carmi M, Fine M, Hoegh-Guldberg O (2010). Increasing the accuracy of surface area estimation using single wax dipping of coral fragments. Coral Reefs.

[44] Jones RJ, Kildea T, Hoegh-Guldberg O (1999). PAM chlorophyll fluorometry: a new in situ technique for stress assessment in scleractinian corals, used to examine the effects of cyanide from cyanide fishing. Mar. Pollut. Bull..

[45] Jones R (2005). The ecotoxicological effects of photosystem II herbicides on corals. Mar. Pollut. Bull..

[46] Davies PS (1989). Short-term growth measurements of corals using an accurate buoyant weighing technique. Mar. Biol..

[47] Aguiar RB, Dickel OE, Cunha RW, Monserrat JM, Barros DM, Martinez PE (2008). Estradiol valerate and tibolone: effects upon brain oxidative stress and blood biochemistry during aging in female rats. Biogerontology.

[48] Oakes KD, van der Kraak GJ (2003). Utility of the TBARS assay in detecting oxidative stress in white sucker (*Catostomus commersoni*) populations exposed to pulp mill effluent. Aquat. Toxicol..

[49] Huang D, Ou B, Prior RL (2005). The chemistry behind antioxidant capacity assays. J. Agric. Food. Chem..

[50] Sokolova IM, Frederich M, Bagwe R, Lanning G, Sukhotin AA (2012). Energy homeostasis as an integrative tool for assessing limits of envirnmental stress tolerance in aquatic organisms. Mar. Environ. Res..

[51] Underwood AJ (1997). Experiments in Ecology: Their Logical Design and Interpretation Using Analysis of Variance.

[52] Halliwell B (2007). Biochemistry of oxidative stress. Biochem. Soc. Trans..

[53] Havaux M, Niyogi KK (1999). The violaxanthin cycle protects plants from photooxidative damage by more than one mechanism. Proc. Natl. Acad. Sci. USA.

[54] Tardy F, Havaux M (1997). Thylakoid membrane fluidity and thermostability during the operation of the xanthophyll cycle in higher-plant chloroplasts. Biochim. Biophys. Acta..

[55] Downs CA, Mueller E, Phillips S, Fauth JE, Woodley CM (2000). A molecular biomarker system for assessing the health of coral (*Montastrea faveolata*) during heat stress. Mar. Biotechnol..

[56] Krueger T (2015). Differential coral bleaching—contrasting the activity and response of enzymatic antioxidants in symbiotic partners under thermal stress. Comp. Biochem. Physiol. Part A: Mol. Integ. Physiol..

[57] Marangoni LFB (2019). Oxidative stress biomarkers as potential tools in reef degradation monitoring: a study case in a South Atlantic reef under influence of the 2015–2016 El Niño/Southern Oscillation (ENSO). Ecol. Ind..

[58] Morris LA, Voolstra CR, Quigley KM, Bourne DG, Bay LK (2019). Nutrient availability and metabolism affect the stability of coral-Symbiodiniaceae Symbioses. Trends Microbiol..

[59] Axenov-Gribanov DV, Bedulina DS, Shatilina ZM, Lubyaga YA, Vereshchagina KP, Timofeyev MA (2013). A cellular and metabolic assessment of the thermal stress responses in the endemic gastropod Benedictia limnaeoides ongurensis from Lake Baikal. Comp. Biochem. Physiol. Part B..

[60] Larade S, Storey KB, Storey KB, Storey JM (2002). A profile of metabolic responses to anoxia in marine invertebrates. Sensing, Signaling and Cell Adaptation.

[61] Philip A, Macdonald AL, Watt PW (2005). Lactate—a signal coordinating cell and systemic function. J. Exp. Biol..

[62] Riobò NA, Clementi E, Melani M, Boveris A, Cadenas E, Moncada S, Poderoso JJ (2001). Nitric oxide inhibits mitochondrial NADH:ubiquinone reductase activity through peroxynitrite formation. Biochem. J..

[63] Wang Y, Ruby EG (2013). The roles of NO in microbial symbioses. Cell. Microbiol..

[64] Higuchi T, Yuyama I, Nakamura T (2015). The combined effects of nitrate with high temperature and high light intensity on coral bleaching and antioxidant enzyme activities. Reg. S. Mar. Sci..

[65] Muscatine L, Porter JW (1977). Reef corals-mutualistic symbioses adapted to nutrient-poor environments. Bioscience.

[66] Ezzat L, Maguer J-F, Grover R, Ferrier-Pagès C (2015). New insights into carbon acquisition and exchanges within the coral-dinoflagellate symbiosis under NH^4+^ and NO^3^^−^ supply. Proc. R. Soc. B..

[67] Cunning R, Baker AC (2013). Excess algal symbionts increase the susceptibility of reef corals to bleaching. Nat. Clim. Change.

[68] Meyer JL, Schultz ET (1985). Migrating haemulid fishes as a source of nutrients and organic matter on coral reefs. Limnol. Oceanogr..

